# Impact of O-H···π Hydrogen Bond on IR and NMR Parameters of Cannabidiol: Theoretical and Experimental Study

**DOI:** 10.3390/molecules30122591

**Published:** 2025-06-14

**Authors:** Aneta Buczek, Kacper Rzepiela, Teobald Kupka, Małgorzata A. Broda

**Affiliations:** Faculty of Chemistry and Pharmacy, University of Opole, 48, Oleska Street, 45-052 Opole, Poland; 119680@student.uni.opole.pl (K.R.); teobald@uni.opole.pl (T.K.); broda@uni.opole.pl (M.A.B.)

**Keywords:** cannabidiol, IR, NMR, DFT, conformational properties, intramolecular H-bond

## Abstract

This study investigates the influence of weak hydrogen bonds on the conformational properties and spectral characteristics of cannabidiol (CBD). Using a combination of FTIR and NMR spectroscopy, we analyze the effects of intramolecular hydrogen bonding, particularly the O-H∙∙∙π interactions, on the molecular behavior of CBD in chloroform solution. FTIR spectra reveal distinct ν_s_(O-H) stretching bands at 3603 cm^−1^ and 3425 cm^−1^, corresponding to free and hydrogen-bonded -OH groups, respectively, with experimental results aligning closely with computational data for CBD conformers. Notably, conformer **1a** predominates in solution, with weaker hydrogen bonding observed for the -OH(B) group compared to -OH(A). Additionally, the formation of -OH∙∙∙π hydrogen bonds affects key vibrational bands in the 1700–1300 cm^−1^ region. NMR analysis shows significant shifts in proton and carbon signals, emphasizing the influence of hydrogen bonding on CBD’s electronic environment. The observed changes in coupling constants, although subtle, further highlight the impact of these interactions on spin–spin coupling. Overall, these findings provide deeper insights into the structural and electronic factors governing CBD’s behavior in solution, offering a basis for future studies on hydrogen bonding in biomolecules and their pharmacological implications.

## 1. Introduction

**Figure 1:**
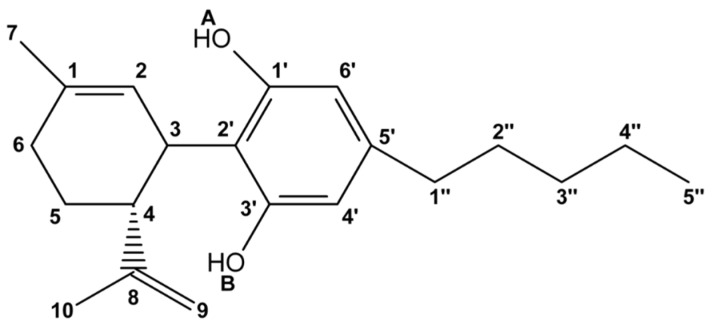
Chemical formula with atom numbering of cannabidiol (CBD).

Cannabidiol (CBD, Figure 1) was first discovered by American chemist Roger Adams, who successfully isolated the compound from wild hemp flowers in Minnesota in 1940 [1,2]. This non-psychoactive cannabinoid [3] exhibits significant biological properties, including anti-inflammatory [4,5,6], antibacterial, antiviral, antioxidative [7,8,9], and anxiolytic effects. It is widely recognized for its therapeutic applications in the treatment of epilepsy [10,11,12] and schizophrenia [13]. Additionally, cannabidiol is used in managing various types of cancer and in palliative care to prevent nausea, vomiting, insomnia, and severe chronic pain [14].

The structural complexity of CBD, which includes two hydroxyl groups (-OH) and a polycyclic structure, makes this molecule an ideal candidate for spectroscopic studies aimed at understanding its conformation and molecular interactions. While the IR spectra of CBD in solution and in solid state have been characterized in several studies [15,16,17,18,19], the role of intramolecular interactions, particularly hydrogen bonding, in shaping the spectroscopic properties of CBD remains underexplored. Hydrogen bonding plays a crucial role in molecular recognition processes, particularly in the binding of ligands to proteins. The presence and orientation of intramolecular hydrogen bonds, such as the O–H···π interaction observed in CBD conformer 1a, can influence the molecular rigidity and spatial arrangement of functional groups critical for receptor binding. For example, studies have shown that conformationally restricted cannabinoids can exhibit altered binding affinities toward CB1 and CB2 receptors, as well as nuclear receptors such as PPARγ [20,21]. In our previous work [22], we demonstrated that CBD adopts a specific conformation in a solution that is stabilized by intramolecular hydrogen bonds between the hydroxyl groups (-OH) and the π-electron system of the C=C double bonds (denoted as OH∙∙∙π interactions) [22]. These interactions are known to influence the spatial arrangement of the molecule [23,24,25], potentially altering the electronic and vibrational properties that are captured in the IR spectra. It is important to note that the crystallographic form of CBD consists of a dimer, in which an intermolecular hydrogen bond (O-H∙∙∙O) is formed between the hydrogen atom of the -OH(B) group of the first molecule and the oxygen atom of the -OH(A) group of the second molecule, as demonstrated in the study [26]. The presence of intermolecular hydrogen bonding may influence the observed shifts in vibrational frequencies.

Molecular modeling of NMR parameters is currently an indispensable tool and an efficient support for deriving the structure of many natural compounds [27,28]. Given its sensitivity, speed, and information delivery capabilities, most hemp extracts are analyzed using ^1^H NMR spectroscopy. For instance, Barthlott et al. [29] reported on the screening of cannabinoids in CBD oils using quantitative ^1^H NMR spectroscopy. They emphasized that this technique is fast and capable of detecting and determining hemp metabolites from an initial, complex organic matrix without requiring separation or sample preparation. At temperatures of 12 °C, 27 °C, and 42 °C, they observed a gradual collapse of two aromatic signals (H4′ and H6′) and broadening of another peak, attributed to the -OH(A) proton. These changes were associated with intramolecular dynamic effects in CBD fragments, enhanced by temperature. This dynamic process is probably due to the restricted rotation of the aromatic ring around a C3-C2′single bond.

^13^C NMR spectroscopy requires more sample material, which explains the limited number of studies involving CBD and related molecules. Recently, Marchetti et al. [30] conducted systematic ^1^H and ^13^C NMR studies of non-psychoactive cannabinoids from fiber-type *Cannabis sativa* L. (hemp) extracts. They compared the obtained spectra with several recorded pure cannabinoid samples as references. The analytical potential of NMR techniques was demonstrated by presenting typical ^1^H, ^13^C, HSQC, and HMBC spectra of a Santhica extract. The authors demonstrated, for the first time, the competitive potential of quantitative ^13^C NMR compared to the traditional HPLC technique for analyzing several hemp components. In 2024, Congcong Yu et al. [31] proposed a certified reference material for cannabidiol. They performed combined quantitative tests of CBD using several analytical methods: ultraviolet absorption spectroscopy (UV), infrared spectroscopy (IR), mass spectrometry (MS), nuclear magnetic resonance spectroscopy (NMR), and differential scanning calorimetry (DSC). The reported proton and carbon chemical shifts closely matched those previously reported by Marchetti et al. [30]. Colella et al. [32] also utilized proton and carbon NMR in the metabolic analysis of natural extracts from *Cannabis sativa*. They discussed both one- and two-dimensional spectra (1D and 2D), as well as certain proton-proton coupling constants. Ohtsuki et al. [33] combined liquid-liquid-based separation techniques and NMR analysis, concluding that quantum mechanical calculations of NMR parameters play a crucial role in analyzing natural product compositions across a wide range of concentrations.

Wood et al. [34] reported the complete ^1^H and ^13^C NMR assignments of cannabicitran and evaluated the performance of a combination of theoretically studied geometry optimization and subsequent gauge independent atom orbital (GIAO) NMR calculations in the gas phase and chloroform approximated by polarized model of solvent (PCM). The use of the PCM model of chloroform showed no improvement. However, it is well known that the choice of functional and basis set in DFT calculations significantly affects the accuracy of predicted NMR parameters [35].

Several authors reported on detailed analysis of NMR and IR spectra, supported by theoretical calculations, of complex natural products [36,37,38]. Other studies [39] have investigated the impact of solvent effects on the spectroscopic properties of cannabinoid derivatives and reported that NMR chemical shifts for carbon atoms can vary by up to 7 ppm due to solvent effect. On the other hand, the changes in NMR parameters, including chemical shifts and J-couplings, can be influenced by the strength and nature of intramolecular hydrogen bonds. These interactions modify the electronic environment of nuclei, leading to variations in shielding and indirect spin–spin coupling constants, which play a key role in determining molecular conformation and stability. In the study by Denhez et al. [40], the effect of intramolecular hydrogen bonding on the conformational stability of cannabidiol derivatives was investigated using NMR, XRD, and DFT calculations. The results indicate that the conformation is influenced by the type of hydrogen bond formed, which, in turn, depends on the choice of solvent. It is important to mention that CBD has low solubility in water but is well soluble in organic solvents, such as chloroform, ethanol, and hexane. Recent research has shown that the poor solubility of CBD in water is attributed to the formation of aggregates, which further influences its physicochemical properties and bioavailability [22].

In this study, we focus on the spectroscopic properties of CBD in chloroform solution, using both IR and NMR spectroscopy to probe its conformation and the potential influence of OH∙∙∙π hydrogen bonds on its spectral parameters. By comparing experimental data with theoretical calculations, we aim to gain new insights into the conformational behavior of CBD and the role of intramolecular interactions in modulating its spectroscopic characteristics. Finally, we explored the possibility of experimental verification of the presence of the two theoretically predicted most stable CBD conformers in the gas phase and chloroform by comparing the root-mean-square (RMS) deviations between DFT-calculated chemical shifts and experimental values.

## 2. Results and Discussion

### 2.1. DFT Conformational Analysis

**Figure 2:**
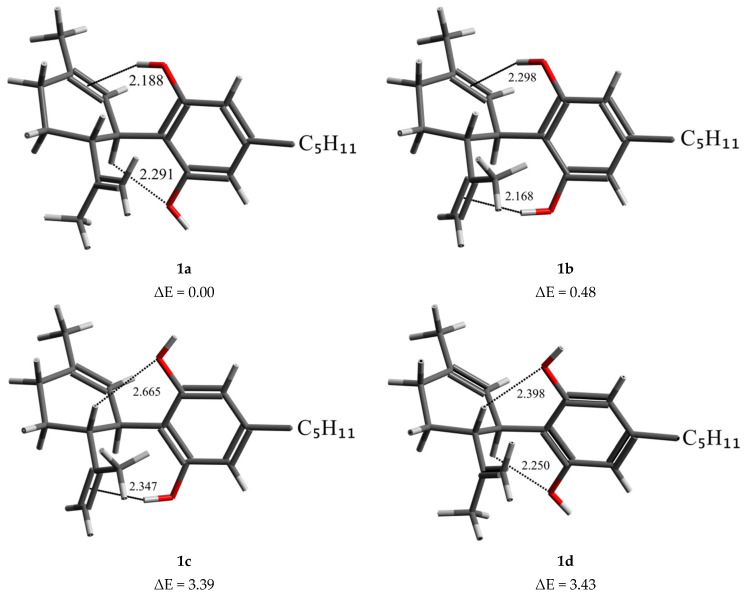
Structures of diequatorial CBD conformers with the lowest energies (relative energies ΔE in kcal/mol) in four groups differing in OH group settings, calculated with MP2/6-311++G**//B3LYP-GD3BJ/6-311++G** method in chloroform. Hydrogen bonds are marked by dot lines, and the distances are given in (Å).

Based on our previous studies [22], it is known that the CBD molecule, in the gas phase and water, can adopt either a diequatorial (**1a**–**1d**) or diaxial (**2a**–**2d**) conformation, depending on the arrangement of substituents at the 3rd and 4th carbon atoms of the limonene ring (Figure 1). These two groups are further divided into four subgroups based on the orientation of hydroxyl groups attached to the aromatic ring of the CBD molecule. In this study, we investigate the conformational and spectroscopic properties of cannabidiol in chloroform, a solvent whose dielectric constant approximates the electrostatic environment within protein interiors. Figure 2 presents the lowest-energy CBD diequatorial conformers from each subgroup, calculated using the MP2/6-311++G**//B3LYP-GD3BJ/6-311++G** method in chloroform, along with their corresponding energy values. The lowest-energy conformer, **1a**, is stabilized by two hydrogen bonds: one O-H∙∙∙π and one C-H∙∙∙O, formed by two hydroxyl groups (O-H(A) and O-H(B) respectively), where the first one acts as a proton donor and the second as a proton acceptor. The next conformer, **1b**, with an energy higher by only 0.48 kcal/mol, is stabilized by two O-H∙∙∙π hydrogen bonds. In **1c** and **1d** conformers with significantly higher energy, 3.39 and 3.43 kcal/mol (see Table 1), the O-H(A) group acts as a proton acceptor, forming C(4)-H∙∙∙O-H(A) hydrogen bond. This means that the energetic order of the CBD conformers is determined primarily by the O-H(A)∙∙∙π interaction. The diaxial conformers have much higher energies, and the analysis of their conformational preferences is presented in the supplement (Figure S1, Table S3). Based on the MP2//DFT calculation results presented above, it can be assumed that CBD in chloroform exists as two diequatorial (**1a** and **1b**) conformers that are in equilibrium.

### 2.2. FTIR Spectra

**Figure 3:**
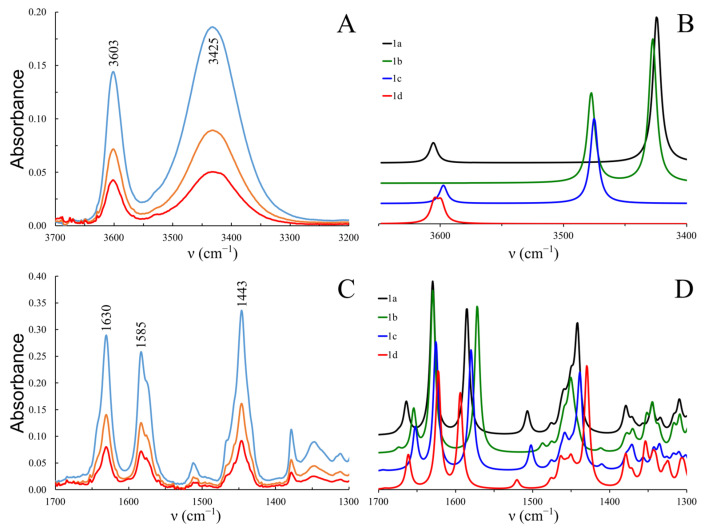
The IR spectra of CBD: (**A**,**B**) (the OH stretching region) and (**C**,**D**) (region below 1700 cm^−1^). (**A**,**C**): experimental spectra in chloroform solution, in three different concentrations ranging from 2.3 × 10^−3^ and 9.3 × 10^−3^ mol L^−1^; (**B**,**D**): spectra calculated with B3LYP-GD3BJ/6-311++G** method in chloroform for the lowest diequatorial CBD conformers (**1a**–**1d**), scaling factors: 0.938 for OH stretching region and 0.976 for 1300–1700 cm^−1^.

Figure 3A presents the ν_s_(O-H) stretching region of the experimental FTIR spectra for cannabidiol (CBD) in chloroform solution. Two distinct absorption bands are observed at 3603 cm^−1^ and 3425 cm^−1^. The band at 3603 cm^−1^ is sharp and is attributed to the stretching vibration of the free -OH group, while the band at 3425 cm^−1^ corresponds to the -OH group engaged in intramolecular hydrogen bonding. The spectra shown correspond to CBD solutions with concentrations ranging from 2.3 × 10^−3^ and 9.3 × 10^−3^ mol L^−1^. Notably, no shifts or changes in the shape of these bands are observed with dilution, suggesting that these bands arise from the monomeric form of CBD.

Figure 3B presents the theoretically calculated vibrational spectra for the νs(O-H) stretching region of the four CBD conformers (**1a**–**1d**), which differ in the orientation of their -OH groups. The calculated spectrum for conformer **1a** exhibits excellent agreement with the experimental data, indicating that this conformation predominates in chloroform solution. The shift to lower frequencies observed in the stretching vibration band of the -OH(B) group, which forms a hydrogen bond with the C8=C9 π-electrons (Δν = 130 cm^−1^), is notably smaller than that of the -OH(A) group, which is bound to the C1=C2 double bond (Δν = 175 cm^−1^). This difference suggests that the interaction involving the -OH(B) group is weaker than that formed by -OH(A). This observation is in agreement with the previously conducted conformational analysis of cannabidiol.

The formation of -OH∙∙∙π hydrogen bonds also influences the position of several bands in the 1700–1300 cm^−1^ region. Figure 3D presents this spectral range calculated for four CBD conformers. The band at 1650–1670 cm^−1^ corresponds to the stretching vibrations of the C8=C9 bond. In conformers **1b** and **1c**, where the -OH(B)∙∙∙π interaction is present, this band is shifted approximately 10 cm^−1^ lower than in conformers **1a** and **1d**, which are not stabilized by this interaction. Additionally, the orientation of the -OH groups notably affects the position of the skeletal vibration bands of the aromatic ring, observed around 1630, 1585, and 1440 cm^−1^ (Figure 3C).

### 2.3. Experimental NMR Spectra of CBD

Intramolecular hydrogen bonds, including O-H∙∙∙π interactions, can cause significant shifts in NMR spectra. The presence of such hydrogen bonds can lead to downfield shifts in the ^1^H NMR spectrum, which indirectly affects the ^13^C NMR chemical shifts due to changes in the electronic environment around the carbon atoms [41]. The size of this ^1^H NMR shift correlates with the strength of the hydrogen bonds.

**Figure 4:**
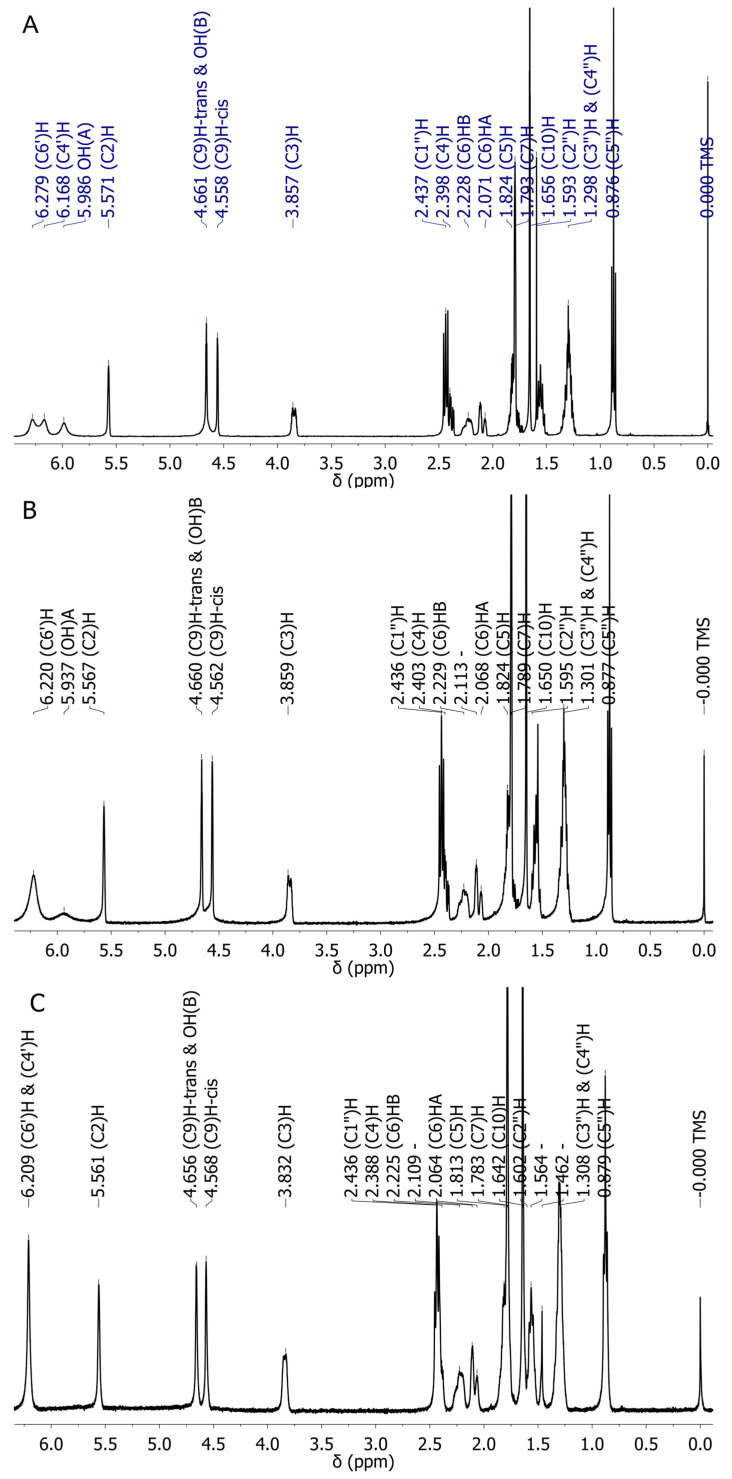
The ^1^H NMR spectrum of CBD in CDCl_3_ at 20 °C (**A**), 30 °C (**B**) and 50 °C (**C**).

The ^1^H NMR spectrum of CBD in CDCl_3_ at 20 °C, 30 °C and 50 °C is shown in Figure 4A–C. Individual peaks are assigned according to earlier works [29,32,42,43]. It is important to notice that at 20 °C, the three peaks in the aromatic region of the spectrum are fairly broad and sharpen at 50 °C. This clearly indicates the presence of a dynamic process, probably due to the relatively fast exchange between conformers in the NMR time scale. Experimental and available literature data of ^1^H chemical shifts of CBD were compared with theoretically predicted values for eight conformers of CBD (four diequatorial and four diaxial; see Table 2 and Table S4). Analysis of the data in Table 2 clearly shows that the chemical shift from the OH(A) group proton depends on whether this group is involved in the OH∙∙∙π bond (6.6 ppm) or participates in the CH∙∙∙O interaction (4.4 ppm). For the OH(B) group, the analogous effect is much smaller (5.8 vs. 4.5 ppm) because this group forms a weaker H-bond. A comparison of the chemical shifts of both OH groups with experimental values suggests that the OH(A) group is involved in the OH∙∙∙π while the OH(B) group is in the C-H∙∙∙O interaction.

The RMS values for theoretical proton data indicate that the smallest difference between the theoretically obtained chemical shifts and our experimental values are observed for the two lowest energy diequatorial conformers (0.37 and 0.34 for **1a** and **1b**, respectively).

This indicates that this compound prefers a structure in which the O-H (A)∙∙∙π hydrogen bond occurs. For conformer **1a**, the largest differences between the experimental and the calculated chemical shift values are observed for the protons of the C(9)-H group, which may be related to the mobility of the phenyl group.

**Figure 5:**
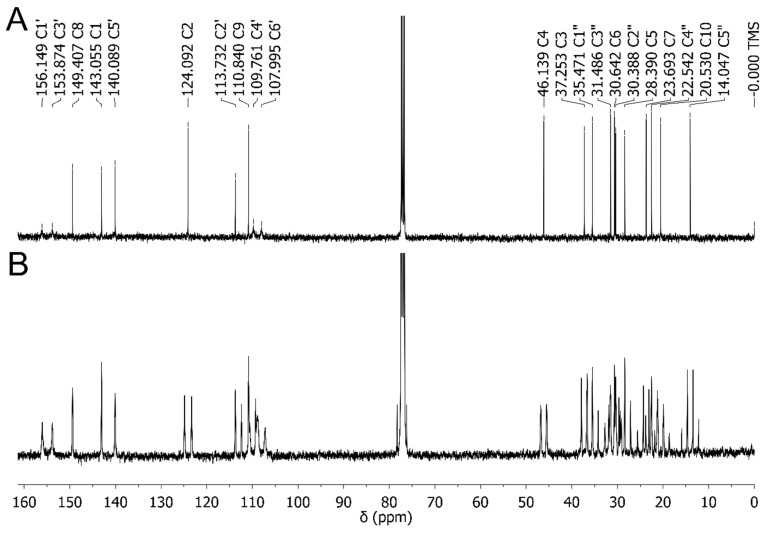
The (**A**) ^13^C (−^1^H) and (**B**) ^13^C (+^1^H) NMR spectra of CBD in CDCl_3_.

The ^13^C (−^1^H) and (+^1^H) spectra are shown in Figure 5A,B, respectively. A typical C-13 NMR spectrum is apparent from Figure 5A, and it agrees with earlier reports [29,32,42,43]. However, the proton–coupled spectrum of CBD was not reported in the literature yet (see Figure 5B). Obviously, the S/N ratio for the latter spectrum is significantly lower, and the accurate determination of several small couplings could be inaccurate. Furthermore, overlapping of some peaks enables only approximate determination of coupling constants. The enlarged aliphatic and aromatic parts of ^13^C (−^1^H) and (+^1^H) spectra are shown in Supplementary Materials (Figure S2). For rigid molecules, the conformation may have little effect on the ^13^C chemical shifts. However, for flexible or cyclic molecules, conformational changes can lead to noticeable shifts in the ^13^C NMR spectrum, and these shifts can be used to infer structural details about the molecule. For example, the chemical shift for the C1 carbon atom in the case of the OH(A)∙∙∙π interaction is 147 ppm (Table 3, for **1a** and **1b** conformers), and in the absence of this interaction, it is approximately 136 ppm. The experimentally determined value for this atom is 143 ppm, which indicates the occurrence of the OH(A)∙∙∙π interaction in CBD conformers in chloroform. The next carbon atoms for which we observe a strong dependence on the adopted conformation are C8 and C10 atoms. For conformers with C3-H∙∙∙OH(B) interactions, the chemical shifts for these atoms are 155 ppm and 20 ppm, respectively, which is very similar to the experimentally observed values. So, despite the fact that RMS values for carbon chemical shifts are relatively large (4.3 to 7.8 ppm), it is the lowest value (4.25 ppm) for the lowest energy conformer according to the DFT results. To sum up the above facts, a detailed analysis of ^1^H and ^13^C chemical shifts indicates that CBD in chloroform occurs in the form of the **1a** conformer, which is stabilized by OH(A)∙∙∙π and C3-H∙∙∙OH(B) interactions.

### 2.4. Indirect Spin–Spin Coupling Constants (SSCCs) of CBD Conformers

Several functionals have been shown to work well for calculating SSCC [44], but generally, the best choice depends on the specific system being studied. Therefore, for conformer **1a**, we compared the coupling constants calculated using the three functionals most popular for this type of calculation: PBE0, B3LYP, and CAM-B3LYP. Proton–proton J-couplings calculated through 2–5 bonds are collected in Table 4 and compared with available literature data. Comparing the RMS values for **1a** in a vacuum, it is clear that B3LYP performs the best, PBE0 is the second-best one, and CAM-B3LYP yields the worst results (Table 4). The corresponding RMS values for these functionals are 1.15, 1.27, and 1.3 Hz, respectively. Moreover, it was shown that the difference between the SSCC values calculated in the gas phase and those obtained using the PCM model for chloroform was small. However, the results of calculations in vacuum agree slightly better with the experimental data. Therefore, for the remaining conformers, the calculations were performed using the B3LYP functional in vacuum. Comparing the calculated coupling constants with the experimental values, it is clear that the lowest RMS value is observed for the **1a** conformer, which is consistent with our hypothesis that in solution, we are dealing mainly with conformer **1a**, possibly with some admixture of **1b**.

It is known that hydrogen bond formation can influence spin–spin coupling constants by altering electronic environments, molecular conformations, and distances between nuclei, leading to variations in SSCC values. Analyzing the data collected in Table 4, it can be observed that the formation of the hydrogen bond OH(B)∙∙∙π (in **1a** and **1b** conformers) causes a decrease in the value of ^2^J(H9A, H9B) coupling constant by about 0.8 Hz. Apart from that, we did not observe any such dependencies for the long-range coupling constants. However, in the case of single-bond couplings, the situation is slightly different.

In Table 5 are gathered one-bond SSCC values for selected C-H couplings. The coupling constants are generally overestimated at the B3LYP/aug-cc-pVTZ level of theory in the gas phase, but the deviations are modest. It can be observed that the coupling constant ^1^J(CH) is larger if the C-H∙∙∙O interaction occurs. A higher constant (by about 2 Hz) occurs for the C3H3 group in conformations **1a** and **1d** and for the C4H4 group in conformations **1c** and **1d**. Furthermore, the formation of an OH∙∙∙π hydrogen bond causes an increase of the coupling constant at the methyl group substituted at the double bond by about 1 Hz. This effect is observed for ^1^J (C7H7) and ^1^J (C10H10).

## 3. Materials and Methods

### 3.1. Experimental

***FTIR spectra:*** The analytical grade CHCl_3_ was dried and purified following standard methods. The IR spectra were recorded at 20 °C using a Nicolet (Madison, WI, USA) Nexus spectrometer equipped with a DTGS detector and flushed with dry nitrogen during the measurements. All spectra were recorded at 1 cm^−1^ resolution and averaged using 100 scans. Solvent spectra were obtained under identical conditions and subtracted from the sample spectra. The thickness of the KBr liquid cell was 2.86 mm, and the concentration varied between 2.3 × 10^−3^ and 9.3 × 10^−3^ mol L^−1^. The spectra were analyzed with the GRAMS AI spectroscopy software suite [17]. The number and position of component bands were obtained from second derivatives and by Fourier self-deconvolution techniques as an ‘initial guess’. Next, the accurate band positions were determined by a curve-fitting procedure with a mixed Gauss-Lorentz profile.

***NMR spectra:*** A sample of CBD (about 5 mg in 0.6 mL CDCl_3_, Aldrich, Saint Louis, MO, USA) was measured with a 400 MHz ultra-shield Bruker NMR spectrometer using TMS as an internal standard. No additional sample purification was applied. For proton spectra, 16 scans were averaged at 20, 30 and 50 °C. Carbon-13 spectra decoupled from protons and coupled with protons were measured at room temperature only (needed considerably longer times of measurements).

### 3.2. Computational Details

***Geometry optimization:*** A detailed analysis of the conformational properties of cannabidiol (CBD) in the gas phase and in water was performed in our previous theoretical study [22], using the PCM/B3LYP-D3BJ/6-311++G(d,p) method. CBD conformers were categorized based on their structural differences, leading to the identification of four lowest-energy diequatorial, **1a**–**1d** (Figure 2) and four diaxial, **2a**–**2d** (Figure S1) conformers.

In this study, additional B3LYP-D3BJ/6-311++G** calculations in chloroform were conducted for eight previously found CBD conformers, and their ground state structure was confirmed by the lack of imaginary frequencies. Based on the full optimization of the diaxial and diequatorial conformers, single-point calculations were performed using the B3LYP and MP2 methods, combined with the 6-311++G** and aug-cc-pVTZ basis sets. All calculations were carried out using the Gaussian 16 software package [45] in both vacuum and chloroform. The solvent effect of chloroform was simulated using a self-consistent reaction field (SCRF) based on the polarizable continuum model (PCM) [46].

***IR calculations***: Vibrational modes were predicted using the harmonic approximation, as implemented in Gaussian software [45], with cost-effective density functional theory (DFT) methods. However, these calculations often overestimate experimental data. To improve accuracy, empirical scaling factors have been applied to harmonic frequencies, significantly improving the agreement with observed data [47].

***NMR calculations***: For each CBD conformer, a single-point GIAO NMR calculation was performed to obtain nuclear shielding tensors using the B3LYP/aug-cc-pVTZ method in chloroform modeled by the PCM method. The raw theoretical shielding data were converted to chemical shifts using earlier predicted isotropic shieldings of reference molecules—TMS and benzene—details in Table S1 in the Supplementary Information. The ^1^H and ^13^C nuclear shieldings, calculated at B3LYP/aug-cc-pVTZ level of theory, are shown in Table S2.

The corresponding theoretical chemical shifts (in ppm) for atoms in the aromatic ring and double bonds were calculated as follows:

δ(^13^C(i)) = σ(ref) − σ (i) + 128.5

δ(^1^H(i)) = σ(ref) − σ (i) + 7.26

The remaining chemical shifts were referenced with respect to TMS.

The computed NMR parameters were then compared with experimental results and available literature data. Additionally, spin–spin coupling constants (SSCC), including ^n^J(HH) and ^1^J(CH), were modeled for the lowest-energy conformer using the B3LYP and PBE0 functionals in the gas phase and chloroform. All SSCC values were calculated with a “mixed” option of aug-cc-pVTZ basis set. Our calculated SSCC values for ^n^J(HH) were compared with data available in the literature, whereas the theoretical SSCC values for ^1^J(CH) were compared with values determined from our experimental NMR spectra.

## 4. Conclusions

Our investigation into the influence of weak hydrogen bonds on the conformational properties and spectral parameters of cannabidiol (CBD) has provided significant insights into its molecular behavior. The presence of the O-H∙∙∙π intramolecular hydrogen bond has been identified as a key stabilizing factor for conformer **1a**, with specific hydrogen bonding interactions, such as OH(A)∙∙∙π and C3-H∙∙∙OH(B), exerting notable effects on vibrational frequencies, chemical shifts, and coupling constants.

FTIR analysis of CBD in chloroform solution reveals distinct ν_s_(O-H) stretching bands at 3603 cm^−1^ and 3425 cm^−1^, which correspond to the free and hydrogen-bonded -OH groups, respectively. The experimental FTIR spectra are in excellent agreement with the calculated data for the CBD conformers (**1a**–**1d**), with conformer **1a** predominating in chloroform solution. The observed shift in the ν_s_(O-H) band suggests a weaker hydrogen bond in the -OH(B) group compared to -OH(A), consistent with previous conformational analyses. Furthermore, the formation of -OH∙∙∙π hydrogen bonds influences the 1700–1300 cm^−1^ spectral region, causing shifts in the C8=C9 bond stretching vibrations and aromatic skeletal vibrations.

The proton and carbon NMR shifts, with changes of up to 2 ppm for protons and 10 ppm for carbons, highlight the impact of hydrogen bonding on the electronic environments of CBD conformers. Although the changes in coupling constants are more subtle, with variations of 1–2 Hz, they still provide evidence of the influence of these interactions on spin–spin coupling magnitudes.

Overall, our findings enhance the understanding of the structural and electronic factors that govern the behavior of CBD in solution, emphasizing the critical role of weak hydrogen bonds in determining conformational preferences and NMR spectral characteristics. This work not only advances our knowledge of cannabidiol’s molecular structure but also lays the groundwork for future studies on the role of hydrogen bonding in other biomolecules and its implications for pharmacological properties.

## Figures and Tables

**Table 1:** The relative energies ΔE (in kcal/mol) of the lowest diequatorial CBD conformers calculated with MP2/6-311++G**//B3LYP-GD3BJ/6-311++G** in gas phase and chloroform. Hydrogen bond distances are given in Å.

		Gas Phase		Chloroform	
Conformer	H-Bond Type	ΔE	Distance	ΔE	Distance
**1a**	C3-H…O-H(B)	0.00	2.284	0.00	2.291
	O-H(A)…C1=C2		2.202		2.188
**1b**	O-H(B)…C8=C9	0.49	2.335	0.48	2.298
	O-H(A)…C1=C2		2.185		2.168
**1c**	O-H(B)…C8=C9	3.46	2.374	3.39	2.347
	C4-H…O-H(A)		2.646		2.665
**1d**	C3-H…O-H(B)	3.97	2.257	3.43	2.250
	C4-H…O-H(A)		2.418		2.398

**Table 2:** Calculated ^1^H chemical shifts of diequatorial CBD conformers, using B3LYP/aug-cc-pVTZ method in chloroform, compared with experiment and available literature data.

Atom	1a	1b	1c	1d	Exp. ^a^	Lit. [42]	Lit. [29]	Lit. [43]	Lit. [32]
**(OA)H**	6.60	6.55	4.44	4.42	5.99	5.99	5.95	6.22	
**(OB)H**	4.34	5.78	5.78	4.47	4.66	5.02	4.6		
**(C2)H**	5.40	5.27	5.05	5.11	5.57	5.57	5.57	5.56	5.57
**(C3)H**	4.15	3.76	3.68	4.24	3.86	3.9	3.84	3.86	3.86
**(C4)H**	2.51	2.27	2.50	3.21	2.40	2.4	2.4		2.40
**(C5)H**	1.81	1.88	1.89	1.83	1.82	1.84	1.82		1.78–1.84
**(C6)H**	2.27	2.28	2.19	2.20	H6_a_ = 2.07 H6_b_ = 2.23	2.21	2.09		H6_a_ = 2.05–2.09 H6_b_ = 2.22
**(C7)H**	1.92	1.92	1.81	1.79	1.79	1.79	1.79		1.79
**(C9)H-trans**	4.10	4.78	4.73	4.04	4.66	4.64	4.67	4.66	4.64
**(C9)H-cis**	3.75	4.74	4.64	4.00	4.56	4.54	4.6	4.57	4.53
**(C10)H**	1.88	1.53	1.50	1.88	1.66	1.66	1.65		1.66
**(C4**′**)H**	5.53	5.94	5.96	5.63	6.17	6.16	6.19		6.16
**(C6**′**)H**	5.85	5.98	5.71	5.56	6.28	6.26	6.25		6.26
**(C1** **″** **)H**	2.53	2.59	2.57	2.52	2.44	2.43	2.44		2.43
**(C2** **″** **)H**	1.52	1.56	1.56	1.52	1.59	1.55	1.56		1.52–1.61
**(C3** **″** **)H**	0.82	0.92	0.93	0.85	1.30	1.29	1.3		1.27–1.32
**(C4** **″** **)H**	1.18	1.19	1.19	1.19	1.30	1.29	1.3		
**(C5** **″** **)H**	0.86	0.85	0.86	0.86	0.88	0.88	0.89		0.86–0.88
**RMS**	**0.37**	**0.34**	**0.50**	**0.54**					

^a^ This work.

**Table 3:** Calculated ^13^C chemical shifts of diequatorial CBD conformers, with B3LYP/aug-cc-pVTZ in chloroform solvent, compared with experiment and available literature data.

Atom	1a	1b	1c	1d	Exp. ^a^	Lit. [42]	Lit. [18]
**C1**	147.20	147.46	135.47	137.45	143.06	134.2	
C2	125.88	124.83	128.17	128.23	124.09	127.3	124.14
C3	41.98	50.42	51.07	41.96	37.25	37.5	37.01
C4	53.66	49.69	49.29	51.76	46.14	46.4	
C5	33.30	34.39	34.95	34.51	28.39	31.7	28.35
C6	35.92	35.86	35.56	36.05	30.64	30.7	30.36
C7	26.86	26.93	26.43	26.43	23.69	23.7	23.69
**C8**	155.26	168.10	169.01	158.00	149.41	150.3	
C9	109.19	106.03	103.95	107.03	110.84	110.5	110.81
**C10**	20.76	29.95	29.98	20.35	20.53	19.5	20.30
C1′	159.83	159.86	158.42	157.52	156.15	157.5	
C2′	112.35	114.82	116.23	114.69	113.73	115.9	
C3′	156.83	157.25	158.18	157.26	153.87	150.3	
C4′	105.13	109.98	110.25	102.81	109.76	108.3	107.92
C5′	145.31	145.84	145.15	145.03	140.09	142.7	
C6′	105.58	107.25	104.94	107.70	107.99	108.3	109.56
C1″	41.68	41.38	41.09	41.33	35.47	36.6	35.46
C2″	38.54	38.50	38.69	38.60	30.39	32.0	30.65
C3″	36.69	36.82	36.76	37.67	31.49	32.6	31.48
C4″	29.80	29.85	29.99	29.33	22.54	23.6	22.54
C5″	17.23	17.12	17.05	16.81	14.05	14.4	14.04
**RMS this work**	**4.70**	**6.85**	**7.30**	**5.06**			

^a^ this work.

**Table 4:** Selected SSCC values for H-H couplings calculated at B3LYP, PBE0, and CAM-B3LYP/aug-cc-pVTZ level of theory in the gas phase and chloroform.

	B3LYP		PBE0		CAM-B3LYP	B3LYP			
	**Gas**	**CHCl_3_**	**Gas**	**CHCl_3_**	**Gas**	**Gas**			
**Coupling Constants**	**1a**					**1b**	**1c**	**1d**	**Lit. [33]**
^4^J(H6′ H4′)	1.26	1.27	1.07	1.08	1.04	1.49	1.32	1.12	3.03
^4^J(H2 H6A)	−3.39	−3.40	−3.81	−3.82	−3.69	−3.39	−3.16	−3.20	−1.45
^4^J(H2 H6B)	−1.16	−1.17	−1.44	−1.44	−1.36	−1.20	−1.68	−1.63	1.35
^4^J(H2 H7)	−1.72	−1.12	−1.96	−1.96	−1.89	−1.73	−1.78	−1.78	−1.12
^2^J(H9A H9B)	3.07	3.29	0.96	1.17	2.69	2.15	2.39	3.47	2.13
^3^J(H3 H2)	2.92	2.88	3.28	3.23	3.31	2.84	2.58	2.62	2.85
^3^J(H3 H4)	10.84	10.83	10.33	10.32	11.11	10.85	10.80	11.00	10.28
^5^J(H3 H7)	3.27	3.24	3.32	3.30	3.43	3.22	3.12	3.22	2.49
^2^J(H6B H6A)	−19.20	−19.44	−19.61	−19.85	−19.58	−19.40	−18.66	−18.51	−17.75
^2^J(H5A H5B)	−13.45	−13.53	−14.01	−14.10	−13.61	−13.91	−13.73	−13.19	−12.88
^3^J(H6A H5B)	5.99	5.99	5.69	5.70	6.05	6.09	6.20	6.23	5.21
^3^J(H6A H5A)	12.70	12.70	11.95	11.95	12.89	12.64	12.61	12.63	11.36
^3^J(H6B H5B)	1.97	1.98	1.90	1.91	2.00	1.87	1.84	1.86	2.12
^3^J(H6B H5A)	5.66	5.65	5.34	5.32	5.76	5.64	5.85	5.92	4.94
^4^J(H6A H7)	−1.64	−1.62	−1.85	−1.83	−1.94	−1.65	−1.72	−1.69	−1.84
^4^J(H6B H7)	−0.65	−0.65	−0.83	−0.82	−0.82	−0.65	−0.76	−0.78	−1.25
**RMS(H)**	**1.15**	**1.17**	**1.27**	**1.29**	**1.30**	**1.15**	**1.16**	**1.21**	

**Table 5:** Selected SSCC values for CH couplings calculated at B3LYP/aug-cc-pVTZ level of theory in the gas phase.

Coupling Constants	1a	1b	1c	1d	Exp. in CDCl_3_
^1^J (C2H2)	161.42	162.54	164.14	162.48	155.14
^1^J (C3H3)	135.76	132.57	129.40	132.80	128.22
^1^J (C4H4)	133.66	133.20	135.27	136.23	127.12
^1^J (C5H5)	132.01	133.13	132.23	131.28	127.04
^1^J (C6H6)	130.02	130.18	129.39	129.24	124.75
^1^J (C7H7)	130.36	130.63	129.59	129.39	126.44
^1^J (C9H9)	162.05	161.72	161.37	161.30	154.76
^1^J (C10H10)	130.26	131.51	131.04	130.11	126.00
^1^J (C4′H4′)	159.91	164.00	157.81	158.08	166.94
^1^J (C6′H6′)	164.46	166.68	167.00	160.12	161.33
^1^J (C1″H1″)	130.56	130.67	130.75	130.58	125.80
^1^J (C2″H2″)	130.17	129.98	129.69	129.89	123.54
^1^J (C3″H3″)	128.78	128.71	128.93	128.98	120.19
^1^J (C4″H4″)	128.61	128.54	128.44	128.52	125.44
^1^J (C5″H5″)	129.12	129.09	129.22	129.26	124.54
**RMS**	**5.82**	**5.65**	**6.12**	**5.87**	

## Data Availability

Data are contained within the article and Supplementary Materials.

## Supplementary Materials

The following supporting information can be downloaded at https://www.mdpi.com/article/10.3390/molecules30122591/s1.
