# Supplementary material for: Impact of O-H···π Hydrogen Bond on IR and NMR Parameters of Cannabidiol: Theoretical and Experimental Study
Source: Molecules. 2025 Jun 14;30(12):2591. doi: 10.3390/molecules30122591 (PMC12195696; doi:10.3390/molecules30122591)
Supplement: Supplementary file 1 [file molecules-30-02591-s001.zip › molecules-3684903-supplementary.pdf]

Supplementary Material
for

Impact of O-H···π hydrogen bond on IR and NMR parameters of cannabidiol (CBD): theoretical and experimental study

Aneta Buczek*, Kacper Rzepiela, Małgorzata A. Broda, Teobald Kupka

Faculty of Chemistry and Pharmacy, University of Opole, 48, Oleska Street, 45-052 Opole, Poland

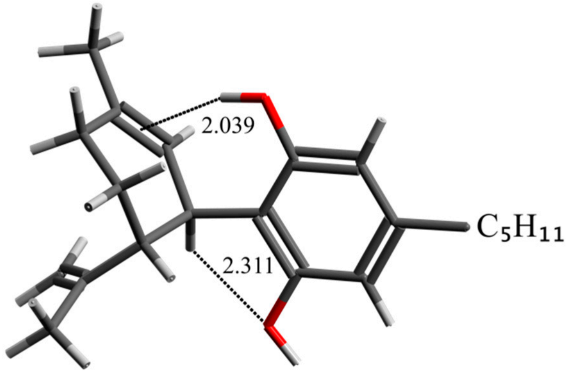

2a
ΔE = 3.77

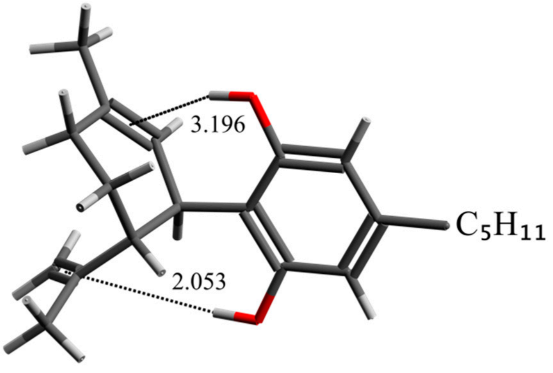

2b
ΔE = 3.84

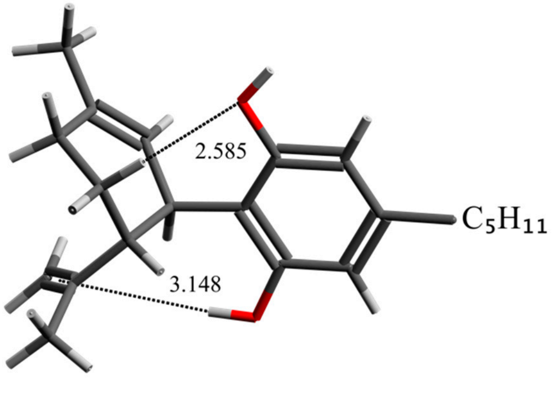

2c
ΔE = 9.16

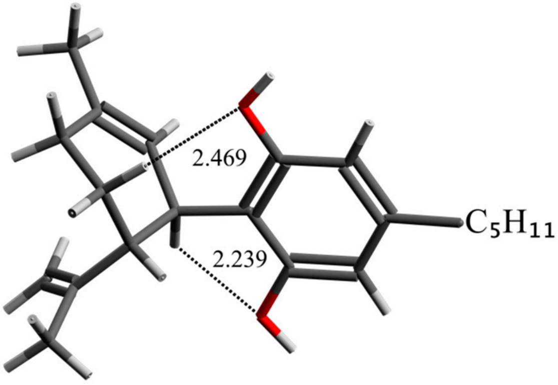

2d
ΔE = 7.83

Figure S1. Structures of diaxial CBD conformers with the lowest energies (relative energies ΔE in kcal/mol, the relative energy was calculated in comparison to the lowest energy of CBD with diequatorial conformation) in four groups differing in OH group settings, calculated with MP2/6-311++G(d,p)//B3LYP-GD3BJ/6-311++G(d,p) method in chloroform. Hydrogen bonds are marked by dot lines and the distances are given in (Å).

Table S1. ^{1}H and ^{13}C nuclear shieldings of TMS and benzene as reference molecules calculated at B3LYP/aug-cc-pVTZ level of theory.

Atom	Gas	$CHCl_3$
TMS		
**C**	184.127	184.512
**H**	31.671	31.665
Benzene		
**C**	49.546	49.326
**H**	24.017	23.907

Table S2. ^{1}H and ^{13}C nuclear shieldings for diequatorial (**1a** - **1d**) and diaxial (**2a** - **2d**) CBD conformers, calculated at B3LYP/aug-cc-pVTZ level of theory in gas phase.

Atom	1a	1b	1c	1d	2a	2b	2c	2d
(OA)H	27.89	26.36	26.10	27.78	27.74	27.26	26.80	27.64
(OB)H	25.40	25.62	27.80	27.77	23.73	24.18	27.95	27.87
(C2)H	25.65	25.78	26.04	25.99	25.39	25.43	25.78	25.81
(C3)H	27.53	27.97	29.46	27.43	27.58	28.25	28.36	27.54
(C4)H	29.13	29.38	28.06	28.56	29.47	29.42	29.53	29.55
(C5)H	29.87	29.81	29.80	29.87	29.83	29.78	29.77	29.72
(C6)H	29.45	29.44	29.52	29.50	29.64	29.66	29.65	29.75
(C7)H	29.78	29.78	29.88	29.89	29.81	29.77	29.88	29.90
(C9)H-trans	26.99	26.40	26.41	27.09	26.54	26.41	26.43	26.52
(C9)H-cis	27.31	26.40	26.49	27.14	26.69	26.65	26.54	26.55
(C10)H	29.81	30.17	30.18	29.79	29.80	29.76	29.77	29.84
(C4’)H	25.23	25.13	25.11	25.71	25.63	25.14	25.08	25.55
(C6’)H	25.74	25.14	25.60	25.66	25.26	25.27	25.72	25.72
(C1”)H	29.18	29.11	29.13	29.19	29.30	29.09	29.28	29.19
(C2”)H	30.16	30.13	30.15	30.18	30.25	30.00	30.24	30.14
(C3”)H	30.82	30.73	30.70	30.80	30.34	30.67	30.34	30.69
(C4”)H	30.50	30.49	30.49	30.50	30.34	30.28	30.34	30.47
(C5”)H	30.81	30.82	30.80	30.80	30.67	30.79	30.66	30.78
C1	33.00	33.25	43.69	42.60	32.81	30.51	40.51	40.40
C2	50.07	50.75	49.06	49.70	51.31	54.28	52.55	52.18
C3	142.51	133.84	132.98	142.10	140.09	138.78	138.41	141.40
C4	131.21	135.03	135.17	132.47	131.57	131.64	131.45	131.00
C5	151.11	150.01	149.40	150.22	158.57	158.80	157.61	158.21
C6	148.36	148.45	148.83	149.15	154.56	153.70	154.91	153.35
C7	157.44	157.45	157.83	157.56	157.44	157.69	156.84	157.01
C8	24.71	10.82	9.80	21.02	22.74	23.15	21.56	22.52
C9	67.37	72.23	73.71	69.83	71.04	70.18	70.37	72.02
C10	163.68	154.44	154.35	163.95	158.96	158.83	158.53	157.62
C1’	21.40	20.49	20.00	18.92	16.13	17.37	20.94	20.00

C2'	66.24	64.52	62.17	61.98	65.76	68.87	64.99	60.54
C3'	17.60	18.17	18.87	20.54	21.10	21.24	18.67	19.17
C4'	71.31	70.29	66.92	72.31	75.73	70.95	71.27	74.83
C5'	33.21	32.54	33.60	34.26	31.22	32.63	31.21	33.96
C6'	73.50	67.31	74.28	73.94	69.49	70.47	74.12	70.00
C1"	142.44	142.78	143.09	142.78	140.15	146.73	140.33	142.69
C2"	145.82	145.82	145.49	145.70	141.11	147.65	141.15	145.46
C3"	147.72	147.46	147.65	147.76	144.18	151.29	143.96	147.87
C4"	154.52	154.41	154.22	154.43	154.11	157.91	154.23	154.27
C5"	167.05	167.14	167.26	167.06	167.03	167.18	167.23	167.31

Table S3. The relative energies ΔE (in kcal/mol) of the lowest diaxial CBD conformer calculated with MP2/6-311++G(d,p)//B3LYP-GD3BJ/6-311++G(d,p) in gas phase and chloroform. Hydrogen bonds distances are given in Å.

	**ΔE**			**Distance**	
**Conformer**	**Gas phase**	**$CHCl_3$**	**H-bond type**	**gas phase**	**$CHCl_3$**
**2a**	3.99	3.77	C3-H···O-H(B)	2.301	2.311
			O-H(A)···C1=C2	2.050	2.039
**2b**	3.87	3.84	O-H(B)···C8=C9	3.183	3.196
			O-H(A)···C1=C2	2.073	2.053
**2c**	9.03	9.16	O-H(B)··· C8=C9	3.137	3.148
			C4-H···O-H(A)	2.593	2.585
**2d**	8.34	7,83	C3-H···O-H(B)	2.239	2.239
			C4-H···O-H(A)	2.459	2.469

Table S4. Calculated ^{1}H chemical shifts of diaxial CBD conformers, with B3LYP/aug-cc-pVTZ in gas phase, compared with experiment and available literature data.

Atom	**2a**	**2b**	**2c**	**2d**	Exp. this work	Lit.1	Lit.2	Lit.3	Lit.4
(OA)H	7.94	7.50	3.72	3.80	5.99	5.99	5.95	6.22	
(OB)H	3.93	4.41	4.87	4.03	4.66	5.02	4.6		
(C2)H	5.78	5.75	5.40	5.36	5.57	5.57	5.57	5.56	5.57
(C3)H	4.09	3.42	3.31	4.13	3.86	3.9	3.84	3.86	3.86
(C4)H	2.20	2.25	2.14	2.12	2.40	2.4	2.4		2.40
(C5)H	1.84	1.89	1.90	1.95	1.82	1.84	1.82		1.78–1.84
(C6)H	2.03	2.01	2.02	1.92	$H6_a$ = 2.07, $H6_b$ = 2.23	2.21	2.09		H6a = 2.05–2.09, H6b = 2.22
(C7)H	1.86	1.90	1.79	1.77	1.79	1.79	1.79		1.79
(C9)H-trans	4.64	4.77	4.75	4.65	4.66	4.64	4.67	4.66	4.64
(C9)H-cis	4.49	4.52	4.63	4.63	4.56	4.54	4.6	4.57	4.53
(C10)H	1.87	1.91	1.90	1.83	1.66	1.66	1.65		1.66
(C4')H	5.54	6.04	6.09	5.63	6.17	6.16	6.19		6.16
(C6')H	5.92	5.90	5.46	5.46	6.28	6.26	6.25		6.26
(C1")H	2.38	2.58	2.39	2.48	2.44	2.43	2.44		2.43
(C2")H	1.42	1.67	1.43	1.53	1.59	1.55	1.56		1.52–1.61
(C3")H	1.33	1.00	1.33	0.98	1.30	1.29	1.3		1.27–1.32
(C4")H	1.33	1.40	1.33	1.20	1.30	1.29	1.3		
(C5")H	1.01	0.88	1.01	0.89	0.88	0.88	0.89		0.86–0.88
RMS exp. this work	0.53	0.41	0.60	0.60					

Table S5. Calculated ^{13}C chemical shifts of diaxial CBD conformers, with B3LYP/aug-cc-pVTZ in vacuum, compared with experiment and available literature data.

Atom	**2b**	**2b**	**2c**	**2d**	Exp. this work	Lit.1	Lit.2
C1	145.24	147.54	137.53	137.65	143.06	134.2	
C2	126.74	123.77	125.50	125.86	124.09	127.3	124.14
C3	44.04	45.35	45.72	42.73	37.25	37.5	37.01
C4	52.56	52.48	52.68	53.13	46.14	46.4	
C5	25.56	25.33	26.52	25.92	28.39	31.7	28.35
C6	29.57	30.43	29.22	30.77	30.64	30.7	30.36
C7	26.69	26.44	27.29	27.12	23.69	23.7	23.69
C8	155.31	154.90	156.48	155.53	149.41	150.3	
C9	107.01	107.87	107.68	106.02	110.84	110.5	110.81
C10	25.17	25.29	25.60	26.50	20.53	19.5	20.30
C1’	161.92	160.68	157.11	158.05	156.15	157.5	
C2’	112.29	109.18	113.05	117.51	113.73	115.9	
C3’	156.95	156.81	159.37	158.88	153.87	150.3	
C4’	102.32	107.09	106.77	103.22	109.76	108.3	107.92
C5’	146.83	145.41	146.84	144.08	140.09	142.7	
C6’	108.56	107.57	103.93	108.05	107.99	108.3	109.56
C1”	43.97	37.40	43.80	41.44	35.47	36.6	35.46
C2”	43.02	36.48	42.98	38.67	30.39	32.0	30.65
C3”	39.95	32.84	40.17	36.26	31.49	32.6	31.48
C4”	30.02	26.22	29.90	29.86	22.54	23.6	22.54
C5”	17.10	16.94	16.89	16.82	14.05	14.4	14.04
RMS this work	5.73	4.10	5.80	4.90			

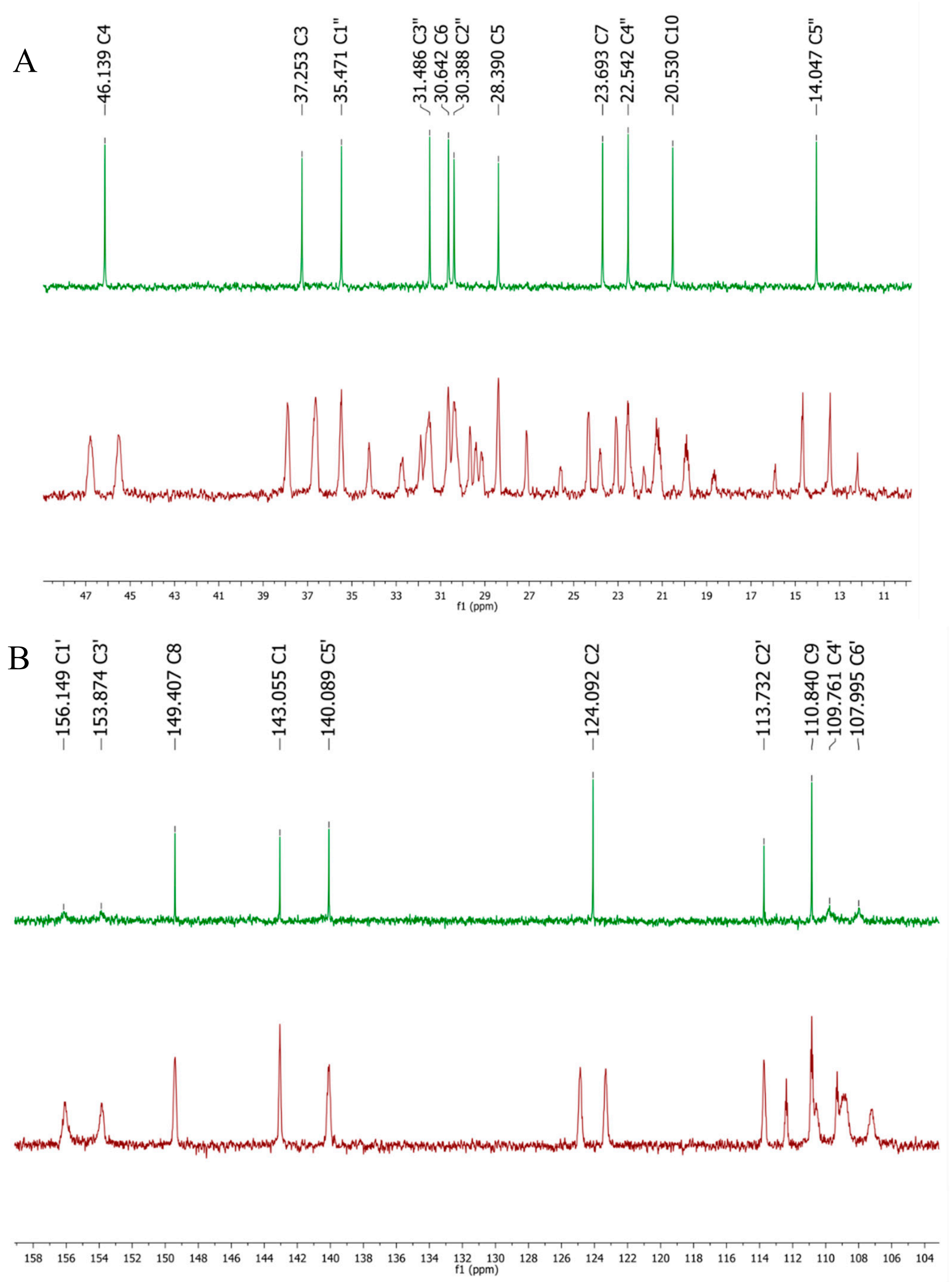

Figure S2. The enlarged aliphatic (A) and aromatic (B) fragments of ^{13}C ($-^1H$) (green) and ($+^1H$) (red) NMR spectrum of CBD in $CDCl_3$.
